# Brain Cells Release Calreticulin That Attracts and Activates Microglia, and Inhibits Amyloid Beta Aggregation and Neurotoxicity

**DOI:** 10.3389/fimmu.2022.859686

**Published:** 2022-04-20

**Authors:** Kyle M. Reid, Emily J. A. Kitchener, Claire A. Butler, Tom O. J. Cockram, Guy C. Brown

**Affiliations:** Department of Biochemistry, University of Cambridge, Cambridge, United Kingdom

**Keywords:** microglia, calreticulin, alarmin, brain, amyloid beta, chaperone, neurotoxicity

## Abstract

Calreticulin is a chaperone, normally found in the endoplasmic reticulum, but can be released by macrophages into the extracellular medium. It is also found in cerebrospinal fluid bound to amyloid beta (Aβ). We investigated whether brain cells release calreticulin, and whether extracellular calreticulin had any effects on microglia and neurons relevant to neuroinflammation and neurodegeneration. We found that microglia release nanomolar levels of calreticulin when inflammatory-activated with lipopolysaccharide, when endoplasmic reticulum stress was induced by tunicamycin, or when cell death was induced by staurosporine, and that neurons release calreticulin when crushed. Addition of nanomolar levels of extracellular calreticulin was found to chemoattract microglia, and activate microglia to release cytokines TNF-α, IL-6 and IL-1β, as well as chemokine (C-C motif) ligand 2. Calreticulin blocked Aβ fibrillization and modified Aβ oligomerization, as measured by thioflavin T fluorescence and transmission electron microscopy. Extracellular calreticulin also altered microglial morphology and proliferation, and prevented Aβ-induced neuronal loss in primary neuron-glial cultures. Thus, calreticulin is released by microglia and neurons, and acts: as an alarmin to recruit and activate microglia, as an extracellular chaperone to prevent Aβ aggregation, and as a neuroprotectant against Aβ neurotoxicity.

## Introduction

Calreticulin is a highly-conserved chaperone that is constitutively expressed in virtually all mammalian cell-types (1). Calreticulin typically resides within the endoplasmic reticulum (ER), where it binds to exposed hydrophobic patches and sugar residues of nascent polypeptides, and functions as a molecular chaperone by refolding misfolded proteins (2). During conditions of apoptosis or ER stress, calreticulin can be translocated to the cell surface (3, 4), where it acts as an ‘eat-me’ signal to local phagocytes, such as macrophages, inducing macrophage phagocytosis of calreticulin-exposed cells by activating the LDL receptor-related protein 1 (LRP1) receptor on phagocytes (3, 5–8). Calreticulin on the surface of phagocytes can also act as a phagocytic co-receptor together with LRP1, enabling LRP1 to be activated by SP-A and SP-D (9) and/or C1q (10, 11). However, calreticulin is a soluble protein, and therefore, once released onto the cell surface, has the potential to be released extracellularly. Indeed, calreticulin has been shown to be secreted by inflammatory-activated macrophages into the extracellular space, where it can opsonise target cells for phagocytosis (12, 13).

Alarmins are endogenous molecules released by necrotic cells and inflamed immune cells, to recruit and activate immune cells, in order to restore homeostasis of damaged tissues (14). Alarmins are therefore also damage-associated molecular patterns (DAMPs), i.e., molecules (such as nucleotides and HMGB1) released by tissue damage to induce an inflammatory response in immune cells to reduce damage. DAMPs were named in analogy with pathogen-associated molecular patterns (PAMP), which are exogenous molecules (such as lipopolysaccharide) released by pathogens, that induce an inflammatory response to combat pathogens. Extracellular calreticulin may also act as a DAMP to activate immune cells (15), although contradictory findings (16) make this unclear. It is not known whether extracellular calreticulin can recruit and activate microglia, so we aimed to test this here.

Extracellular chaperones (such as clusterin) are proteins in the extracellular space that help fold other proteins into a functional or non-pathogenic form (17, 18). This may be particularly important in Alzheimer’s disease, where the extracellular space of the brain becomes clogged up by the aggregation of amyloid beta (Aβ) into extracellular plaques, and extracellular chaperones may potentially prevent this (19). Activated microglia release calreticulin into the culture medium (13), and calreticulin is bound to Aβ in human cerebrospinal fluid (20). Thus, it is possible that calreticulin acts as an extracellular chaperone to keep Aβ in solution or promote its degradation (20, 21). We tested here whether calreticulin could prevent Aβ aggregation into oligomers and fibrils, and if so whether it can prevent Aβ-induced neurotoxicity.

## Materials and Methods

### Materials

All cell culture reagents were from Invitrogen (Paisley, UK), unless otherwise indicated. Culture treatments were procured as follows: staurosporine (from *Streptomyces* sp.) and recombinant human calreticulin were from Abcam (Cambridge, UK); peptide synthesized human amyloid-beta (1-42) was purchased from Anaspec (Fremont, CA); adenosine 5’-triphosphate (ATP) disodium salt hydrate, lipopolysaccharide (LPS; from *Salmonella Enterica* serotype typhimunium), polymyxin B sulfate salts and tunicamycin (from *Streptomyces* sp.) were from Sigma-Aldrich (St Louis, MO, USA); recombinant mouse LRPAP-1 protein was from R&D Systems (Minneapolis, USA).

### Cell Culture and Treatments

The immortalized cell lines BV-2 (ECACC Cat# 0356, RRID: CVCL_0182) and CHME3 (ATCC Cat# CRL-3304, RRID: CVCL_II76) were maintained as previously described (22, 23). Neither cell line is listed as a commonly misidentified cell line by the International Cell Line Authentication Committee. Primary microglial and mixed neuron-glial cultures were respectively prepared from the cortex and cerebellum of 3-5 day old Wistar rats (Charles River, RRID: RGD_2312511), following procedures described elsewhere (24, 25). All animal experiments were approved by the Cambridge University Local Research Ethics Committee and undertaken in accordance with the UK Animals (Scientific Procedures) Act (1986).

Cells were treated as follows: calreticulin was used at 1 nM, 10 nM, 100 nM or 170 nM for 20 hours; 10 nM, 17 nM or 50 nM for 24 hours; 10 nM or 50nM for 48 hours; and 2.5 nM, 10 nM or 50nM for 72 hours as indicated. Monomeric Aβ was used at 250 nM over 72 hours. LPS was added at 100ng/mL over 20 or 24 hours where indicated. Calreticulin or LPS were pre-treated with 10 U/mL polymyxin B for 1 hour where indicated. Polymyxin B was used at 10 U/mL for 20 hours. LRPAP-1 was used at 100 nM for 20 hours. Tunicamycin was used at 0.2 and 2 µg/mL for 24 hours. Staurosporine was used at 10 nM and 100 nM for 24 hours, and at 1000nM for 6 or 24 hours as specified. ATP was added at 10 µM for 24 hours.

### Cell Viability

Cell viability, defined as the percentage of non-necrotic cells, was measured at indicated endpoints by differential dye uptake of propidium iodide (identifying necrotic cells) and Hoechst 33342 (identifying all cells) using a fluorescent microscope (EVOS M5000). Alexa Fluor 488-tagged isolectin B4 was used to identify microglia. Apoptotic cells were recognized as non-necrotic cells with nuclear condensation (identified with Hoechst 33342). For pure microglial cultures, the entire well was imaged, and the number of cells quantified using QuPath (version 0.3.0). Two wells were quantified per experiment, with the number of experimental replicates indicated in the figure legends. For neuronal-glial co-cultures, four microscopic fields were quantified per well, with two wells per experiment, and three experimental replicates.

### Transwell Migration Assay

BV-2 migration was measured by transwell (Boyden) chamber assay using COSTAR 24-well plate 8.0µm pore membrane inserts (Corning). Briefly, 2 x 10^4^ BV-2 cells were seeded into serum-free DMEM on the top chamber membrane. In the bottom chamber, serum-free DMEM, supplemented with calreticulin or ATP, was added. Migration was assessed across 24 hours under incubating conditions (37°C, 5% CO_2_). At experimental endpoints, residual microglia found on top of the membrane were removed using a cotton bud. Microglia that had migrated to the underside of the membrane were fixed in 4% paraformaldehyde, washed in PBS, and stained with Hoechst 33342. Migrated microglia were imaged using the EVOS M5000 (4x Magnification, DAPI Channel).

### Measurement of Calreticulin Release

Calreticulin release from BV-2 microglia and mixed neuron-glia were measured by quantitative western blot. Briefly, 5 x 10^4^ BV-2 cells were cultured in serum-free DMEM and treated accordingly. Neuron-glial culture medium was replaced with PBS supplemented with proteinase inhibitors, and cultures were compressed with the flat end of a syringe plunger for one minute to rupture the cells. Proteins from the conditioned mediums of treated cultures were resolved on NuPAGE 4-12% Bis-Tris gels (Invitrogen) and transferred onto PVDF membranes. Blots were probed with a polyclonal antibody to calreticulin (1:500, EnzoLifeSciences) followed by IRDye^®^ 800CW donkey anti-rabbit IgG secondary antibody (1:5000, Licor). Antibody binding was detected using the LI-COR Odyssey^®^ CL_X_. Quantification of calreticulin loaded was attained by comparing band intensity values for each condition, quantified by Licor Image Studio™, to a standard curve created using 1, 2.5 and 5 ng of recombinant calreticulin loaded within each gel.

Calreticulin release from BV-2 microglia treated with LPS was measured by ELISA. Briefly, 5 x 10^4^ BV-2 cells were cultured in low-serum DMEM. After 24 hours, supernatants were assessed for calreticulin using a calreticulin ELISA (Abbexa), following the supplied protocol. Absorbances were read at 450nm by the FLUOstar OPTIMA spectrophotometer (BMG Labtech, Ortenberg, Germany).

### Measurement of Inflammatory Molecules

Release of inflammatory molecules from BV-2 microglia and primary rat microglia were measured by ELISA. Briefly, 5 x 10^4^ BV-2 cells were seeded in serum-free DMEM, 5 x 10^4^ primary rat microglia were seeded in appropriate medium, and microglia were treated accordingly for 20 hours. Following treatments, supernatants were removed, and protein detection was achieved using mouse ELISAs for TNF-α, IL-6 and CCL2, and a rat ELISA for TNF-α, as per the manufacturer’s instructions (all BioLegend). Absorbances were read at 450nm by a spectrophotometer.

Release of inflammatory molecules from CHME3 microglia were assessed using a custom pre-coated meso scale discovery plex human pro-inflammatory panel 2 (Meso Scale Discovery). Briefly, 3 x 10^4^ CHME3 cells were seeded in serum-free DMEM and treated with calreticulin. After 20 hours, supernatants were removed, and protein detection was achieved by electrochemiluminescence for human TNF-α, IL-6 and IL-1β as per the manufacturer’s instructions.

### Measurement of Endotoxin

Endotoxin (LPS) levels were measured from recombinant calreticulin preparations using the Pierce™ limulus amebocyte lysate (LAL) chromogenic endotoxin quantification kit (Thermo Fisher Scientific), as per manufacturer’s protocol. Endotoxin levels are expressed as endotoxin unit per milliliter (EU/mL).

### Proliferation Assay

2,000 BV-2 cells per well were seeded in DMEM and treated with ± calreticulin. After 24, 48 and 72-hour periods, microglial count and viability were measured through differential dye uptake of Hoechst 33342 and propidium iodide by fluorescence microscopy.

### Amyloid-β Preparation

Peptide synthesized human amyloid-beta 1-42 was prepared as previously described (26). Briefly, Aβ was dissolved in 1,1,1,3,3,3-hexafluoroisopropanol (HFIP, Sigma) and dried under a stream of nitrogen. Prior to the aggregation assay, Aβ was resolubilized by DMSO (Sigma) and resuspended in DMEM.

### *In Vitro* Amyloid-β Aggregation Assay

Aβ aggregation was measured by thioflavin T fluorescence as previously described (27). Briefly, thioflavin T (Sigma) and Aβ (10 µM final concentration for both) were added to DMEM ± 0.1 μM or 1 μM calreticulin. Aβ was let to aggregate at 31.5°C, with orbital shaking for 10 seconds, every 10 minutes and fluorescence (440 nm absorbance, 480 nm emission) was measured at 10 minute intervals within the plate reader. At the end of the assay, aggregates in wells were imaged with a fluorescence microscope (DMI6000; Leica) using 480/40 nm excitation and 527/30 nm emission filters with a 40x objective.

### Preparation of Amyloid-β Oligomers for Transmission Electron Microscopy

To generate oligomers, monomeric Aβ (10 μM) was incubated ± 1 μM calreticulin [18 h at 4°C using a thermocycler (Thermo Fisher Scientific)]. These oligomeric samples (10 μL) were applied to carbonate coated grids for 1 min and negatively stained with 1% uranyl acetate for 1 minute. Micrographs were obtained on a Tecnai G2 transmission electron microscope.

### Statistical Analysis

Statistical analyses were conducted using GraphPad Prism v9. Statistical differences between two groups were analyzed by unpaired and paired t-tests where indicated. Statistical differences between three or more groups were analyzed by one-way repeated measures or mixed model ANOVA followed by Dunnett’s or Šidák multiple comparisons *post-hoc* test. Normality of acquired data was tested by Shapiro-Wilk test. Error bars represent the standard error of the mean of experiments (SEM). p-values refer to the probability of the null hypothesis that the means do not differ. p<0.05 was considered significant, and p≥0.05 not significant.

## Results

### Microglia and Neurons Release Calreticulin

**Figure 1:**
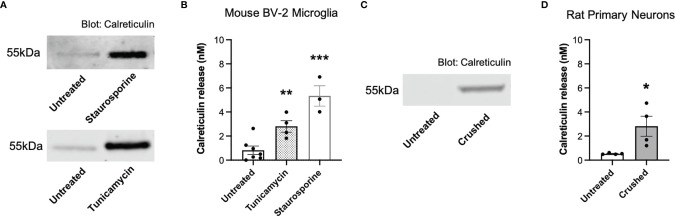
Calreticulin is released from ER-stressed and dying microglia, as well as crushed neurons. **(A, B)** Calreticulin release from mouse BV-2 microglia treated with tunicamycin (2 µg/mL) or staurosporine (1 µM) for 20 hours, measured by western blots. **(A)** Representative blots. **(B)** Calreticulin release from treated BV-2 microglia for all experiments. **(C, D)** Calreticulin release from rat primary neurons before and 1 minute after applying mechanical stress (crushing), measured by western blot. **(C)** Representative blot. **(D)** Calreticulin release from crushed rat primary neurons for all experiments. Calreticulin release was quantified by cross-examining densitometry values of western blot bands to internal recombinant calreticulin standard curves. Data presented as mean calreticulin release values, with error bars representing the SEM of at least 3 independent experiments. Statistical comparisons were made to the untreated control by **(B)** one-way ANOVA and **(D)** paired t-test. *p<0.05, **p<0.01, ***p<0.001.

We have previously shown that primary mouse microglia release calreticulin into the medium when inflammatory activated with lipopolysaccharide, LPS (13), and we confirmed here that BV-2 microglia release calreticulin in response to LPS (**Supplementary Figure 1A**), without inducing cell death (**Supplementary Figure 1B**). We then tested whether BV-2 microglia would release calreticulin, measured by western blots of cell culture supernatants, in various other conditions. Tunicamycin, which induces ER stress by inhibiting protein glycosylation (28), induced significant calreticulin release (**Figures 1A, B**), without inducing significant cell death (**Supplementary Figure 2**). Staurosporine, which induces apoptosis by inhibiting protein phosphatases (29), killed the cells (**Supplementary Figure 2**), and induced release of calreticulin into the medium (**Figures 1A, B**).

We then tested whether necrotic neurons would release calreticulin, and found that crushed cerebellar granule neurons significantly released calreticulin into the culture medium, measured by western blots (**Figures 1C, D**). The reason for crushing the neurons was to test whether calreticulin would be released into the medium when rupturing their plasma membrane. Thus, dying microglia and neurons, and ER-stressed or inflammatory-activated microglia, can release calreticulin, resulting in nanomolar levels in the extracellular culture medium.

### Calreticulin Activates Microglia

**Figure 2:**
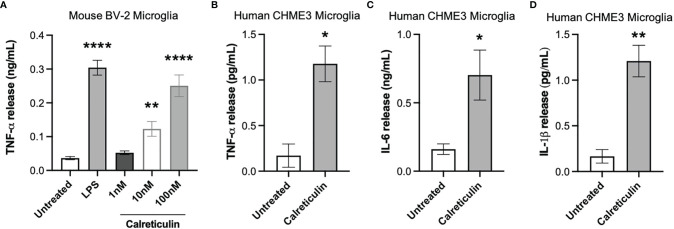
Calreticulin induces cytokine release from mouse and human microglia. **(A)** BV-2 microglia were treated with lipopolysaccharide (LPS, 100 ng/mL) or human, recombinant calreticulin (1 nM, 10 nM or 100 nM) for 20 hours. Supernatants from treated BV-2 were assessed for TNF-α by ELISA. **(B–D)** CHME3 microglia were treated for 20 hours with 170 nM calreticulin (human, recombinant). Supernatants from treated CHME3 were assessed for **(B)** TNF-α, **(C)** IL-6 and **(D)** IL-1β by meso scale discovery. Data presented as mean cytokine release values, with error bars representing the SEM of 3 independent experiments. Statistical comparisons were made to the untreated control by **(A)** one-way ANOVA and **(B–D)** unpaired t-test. *p<0.05, **p<0.01, ****p<0.0001.

Calreticulin is known to be present in human cerebrospinal fluid, but the levels are unknown (20). Having found that microglia and neurons can release nanomolar levels of calreticulin, we tested whether the addition of nanomolar levels of calreticulin can activate microglia to release pro-inflammatory cytokines. Human, recombinant calreticulin was used here and in all subsequent experiments. First, we added 0 nM, 1 nM, 10 nM or 100 nM concentrations of extracellular calreticulin to mouse BV-2 microglia and measured the amount of extracellular TNF-α (tumor necrosis factor alpha) in the medium 20 hours later by ELISA, and compared the release to that induced by the positive control, LPS. Untreated BV-2 microglia released no detectable TNF-α, while 1 nM, 10 nM and 100 nM calreticulin induced the release of 0.05, 0.12 and 0.25 ng/ml TNF-α, and LPS induced the release of 0.35 ng/ml TNF-α (**Figure 2A**). Thus, nanomolar concentrations of calreticulin can induce TNF-α release from BV-2 microglia, at a similar level to that induced by LPS.

As mouse microglia may differ from human microglia (30), we tested whether calreticulin can activate human microglia to release cytokines, using a human microglial cell line, CHME3 [also known as HMC3 (31)]. By meso scale discovery, we measured TNF-α, IL-6 (interleukin 6) and IL-1β release from CHME3 microglia treated for 20 hours with calreticulin. Calreticulin induced significant release of TNF-α, IL-6 and IL-1β from this human microglial cell line (**Figures 2B–D**).

**Figure 3:**
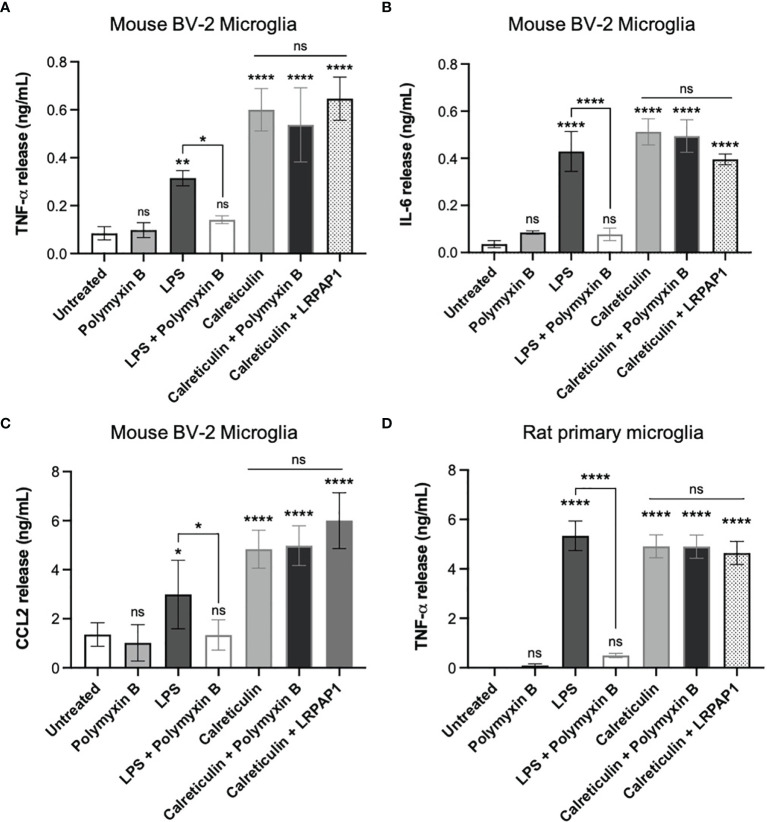
Calreticulin induces cytokine and chemokine release from BV-2 and primary microglia independent of endotoxin and LRP1. BV-2 and rat primary microglia were treated with polymyxin B (10 U/mL), lipopolysaccharide (LPS, 100 ng/mL) ± polymyxin B, and calreticulin (170 nM) ± polymyxin B or LRPAP1 (100 nM) for 20 hours. Supernatants from treated BV-2 were assessed for **(A)** TNF-α, **(B)** IL-6 and **(C)** CCL2 by ELISA. **(D)** Supernatants of treated primary microglia were assessed for TNF-α by ELISA. Data presented as mean cytokine or chemokine release values, with error bars representing the SEM of at least 3 independent experiments. Statistical comparisons were made to the untreated control, or as illustrated by a comparison line, by one-way ANOVA. ns: p≥0.05, *p<0.05, **p<0.01, ****p<0.0001.

As the calreticulin we used was recombinant, and recombinant proteins are potentially contaminated with LPS (32), we tested whether Polymyxin B, which binds and inactivates LPS (33), could prevent the release of TNF-α induced by calreticulin in BV-2 microglia. Polymyxin B prevented the TNF-α release from BV-2 microglia induced by LPS, but had no effect on the TNF-α release induced by calreticulin (**Figure 3A**). This suggests there was little or no LPS contamination of the calreticulin used. This was confirmed by limulus amebocyte lysate (LAL) assay, which measured LPS levels in the calreticulin preparation to be negligible (0.22 ± 0.01 EU/mL (<1 ng of LPS)).

One potential means by which extracellular calreticulin might activate microglia is *via* the receptor LRP1, as this receptor mediates some actions of extracellular calreticulin (34, 35). LRP1 is inhibited by LRPAP1 (LRP-associated protein (36);), so we tested whether added LRPAP1 could prevent calreticulin inducing TNF-α release from BV-2 microglia, but found that it had no effect on this release (**Figure 3A**).

In order to test whether calreticulin can induce the release of other cytokines or chemokines from BV-2 microglia, we added calreticulin to BV-2 microglia and measured IL-6 and CCL2 in the extracellular medium 20 hours later by ELISA. IL-6 is a pro-inflammatory cytokine, and CCL2 (chemokine (C-C motif) ligand 2) is a chemokine, also known as monocyte chemoattractant protein 1 (MCP1). Calreticulin induced the release of both IL-6 and CCL2, and this was not prevented by Polymyxin B or LRPAP1 (**Figures 3B, C**). Thus, calreticulin induces the release of multiple pro-inflammatory cytokines and a chemokine from BV-2 microglia, and this does not appear to be due to LPS contamination or activation of LRP1.

As BV-2 are a cell line, which may diverge from primary cells as a result of transformation and/or mutations (37), we also tested whether calreticulin could induce TNF-α release from primary microglia isolated from rat brain. Calreticulin did induce TNF-α release from primary microglia, and this was not prevented by Polymyxin B or LRPAP1 (**Figure 3D**), just as BV-2 microglia (**Figure 3A**).

We conclude that nanomolar extracellular calreticulin can activate microglia (mouse, rat and human, primary and cell lines), as measured by the release of pro-inflammatory cytokine and chemokines, and this is not mediated by LPS or LRP1.

### Calreticulin Chemoattracts Microglia

**Figure 4:**
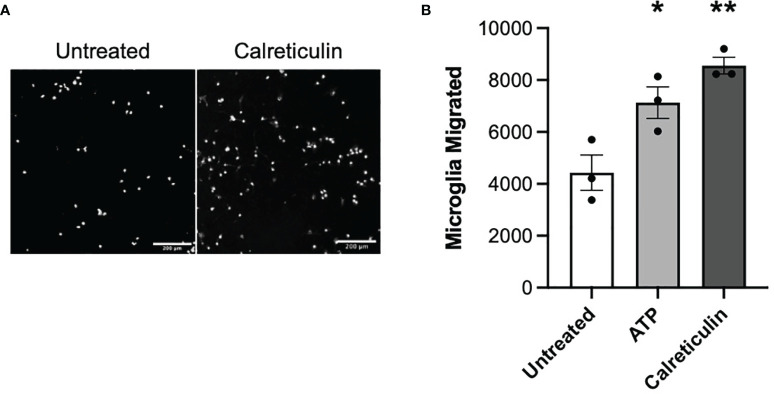
Microglia migrate towards calreticulin. **(A)** Representative images and **(B)** quantified migration of BV-2 microglia migration across a transwell towards 17 nM calreticulin over 24 hours. 10 µM adenosine triphosphate (ATP) acted as the positive control. Microglia were stained with Hoechst 33342. Scale bar = 200 µm. Data presented as mean microglial migration values, with error bars representing the SEM of 3 independent experiments. Statistical comparisons were made to the untreated control by one-way ANOVA. *p<0.05, **p<0.01.

Having found that damaged or activated brain cells release calreticulin, and that added calreticulin can activate microglia, we went on to test whether extracellular calreticulin can do anything else relevant to neuroinflammation and neurodegeneration, in particular whether calreticulin can act as an alarmin, extracellular chaperone or neuroprotectant. If extracellular calreticulin acts as an alarmin, then it should chemoattract microglia. We tested whether extracellular calreticulin can chemoattract microglia by placing BV-2 microglia on top of a transwell (Boyden) chamber and culture medium ± calreticulin or ATP in the lower chamber. ATP is known to chemoattract microglia (38), and acted as a positive control here. After 24 hours, the number of cells that had migrated through the transwell membrane (with 8 µm pores) were counted, and both calreticulin and ATP were found to significantly stimulate microglial migration (**Figures 4A, B**). Thus, extracellular calreticulin at nanomolar concentrations can chemoattract microglia.

### Calreticulin Reduces Microglial Proliferation

**Figure 5:**
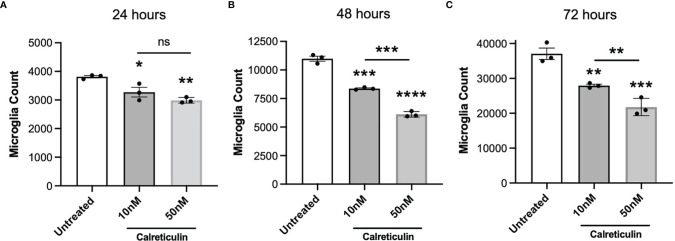
Calreticulin dose-dependently inhibits the proliferation of BV-2 microglia. BV-2 were seeded at a density of 2000 cells/well, then treated with either 0, 10 or 50 nM calreticulin. After incubations for 24 hours **(A)**, 48 hours **(B)** or 72 hours **(C)**, microglia were stained with Hoechst 33342 and microglial cell numbers were quantified by fluorescence microscopy. Data presented as mean microglial cell numbers, with error bars representing the SEM of 3 independent experiments. Statistical comparisons were made to the untreated control, or as illustrated by a comparison line, by one-way ANOVA. ns: p≥0.05, *p<0.05, **p<0.01, ***p<0.001, ****p<0.0001.

If extracellular calreticulin activates microglia, then we might expect it to affect microglial proliferation. Calreticulin can alter the proliferation of a variety of cell types, and is implicated in tumor growth (39), tumor suppression (40, 41), and wound healing (42). We therefore tested whether calreticulin affected the proliferation of microglia. To do this, we seeded the same number of BV-2 cells in culture, to which we added either 0, 10 or 50 nM calreticulin, and then counted the numbers of cells after 24, 48 and 72 hours. Across all timepoints, calreticulin dose-dependently reduced the proliferation of microglia (**Figures 5A–C**), without inducing cell death (**Supplementary Figure 3**). 10 nM calreticulin significantly inhibited proliferation at all times, and 50 nM calreticulin inhibited proliferation more, but, note that even in the presence of 50 nM calreticulin, the BV-2 microglia still proliferated substantially (**Figure 5**). Thus, exogenous calreticulin mildly reduces microglial proliferation.

### Calreticulin Alters Aggregation of Amyloid Beta (Aβ)

**Figure 6:**
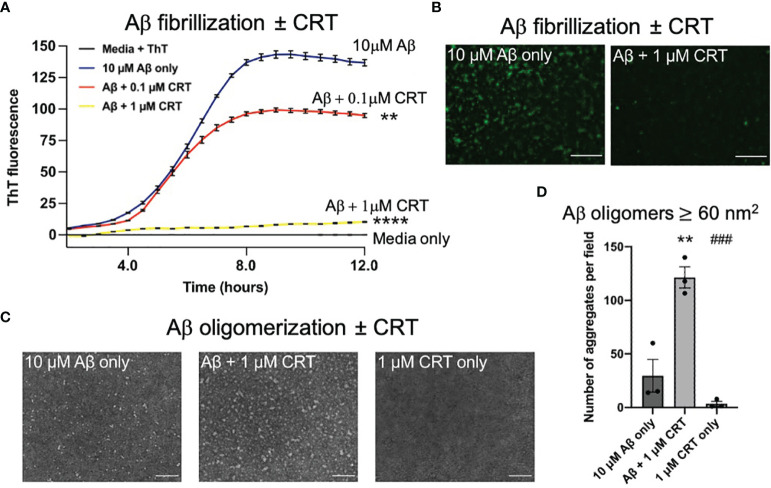
Calreticulin prevents fibrilization and promotes formation of large oligomeric structures of amyloid beta. **(A)** 10 μM of monomeric Aβ was incubated at 31.5°C in DMEM with 10 μM thioflavin T ± 0.1 μM or 1 μM calreticulin (CRT), and the fluorescence recorded over time. **(B)** At the end of the assay, each well was imaged using a fluorescence microscope. Scale bar = 50 μm. **(C)** 10 μM of monomeric Aβ was incubated for 18 h at 4°C ± 1 μM CRT to generate Aβ oligomers imaged by transmission electron microscopy. Representative images shown. Scale bar = 100 nm. **(D)** Quantification of aggregate ≥60 (nm^2^) quantified from **(C)**. Data presented as mean final fluorescence or average aggregate size values, with error bars representing the SEM of at least 3 independent experiments. Statistical comparisons were made by one-way ANOVA. Asterisk (*) indicate significance (**p<0.01, ****p<0.0001) to 10 μM Aβ only. Hash (#) indicate significance (^###^p<0.001) to 10 μM Aβ + 1 μM CRT.

Calreticulin is an intracellular chaperone, which can bind Aβ (21), but it is not known whether calreticulin can act as extracellular chaperone for Aβ, which would be particularly relevant for Alzheimer’s disease. We investigated whether calreticulin affected Aβ aggregation using an *in vitro* fibrillization assay, incubating pure monomeric Aβ with thioflavin T, which becomes fluorescent when bound to β-sheet structures within Aβ aggregates (43). In the absence of calreticulin, Aβ (10 μM) fibrillized with standard lag-phase kinetics at 31.5°C (**Figure 6A**). In the presence of 1 μM calreticulin, fibrillization of Aβ was almost completely prevented (**Figure 6A**). While in the presence of 0.1 μM calreticulin, the kinetics of Aβ fibrillization was little affected, but the maximum fluorescence was reduced (**Figure 6A**). This suggests that calreticulin may help fold Aβ into forms with less β-sheet structure, consistent with calreticulin acting as a chaperone for Aβ. Imaging the Aβ aggregates after the fibrillization assay confirmed that calreticulin reduced the thioflavin T fluorescence of the Aβ aggregates (**Figure 6B**).

As oligomeric Aβ is widely considered to be more neurotoxic than Aβ fibrils (44), we next assessed the effect of calreticulin on Aβ oligomer formation. We incubated 10 μM monomeric Aβ ± 1 μM calreticulin for 18 hours at 4°C [conditions known to result in Aβ oligomers (43)], and imaged the resulting Aβ oligomers by transmission electron microscopy. Aβ oligomerized in the presence of calreticulin resulted in larger oligomeric Aβ structures compared to Aβ oligomers formed in the absence of calreticulin (**Figures 6C, D**).

Taken together, this data suggests that calreticulin inhibits Aβ fibrillization, or folds Aβ into aggregates with less β-sheet structure, and promoted the formation of larger oligomeric structures.

### Calreticulin Prevents Aβ-Induced Neuronal Loss

**Figure 7:**
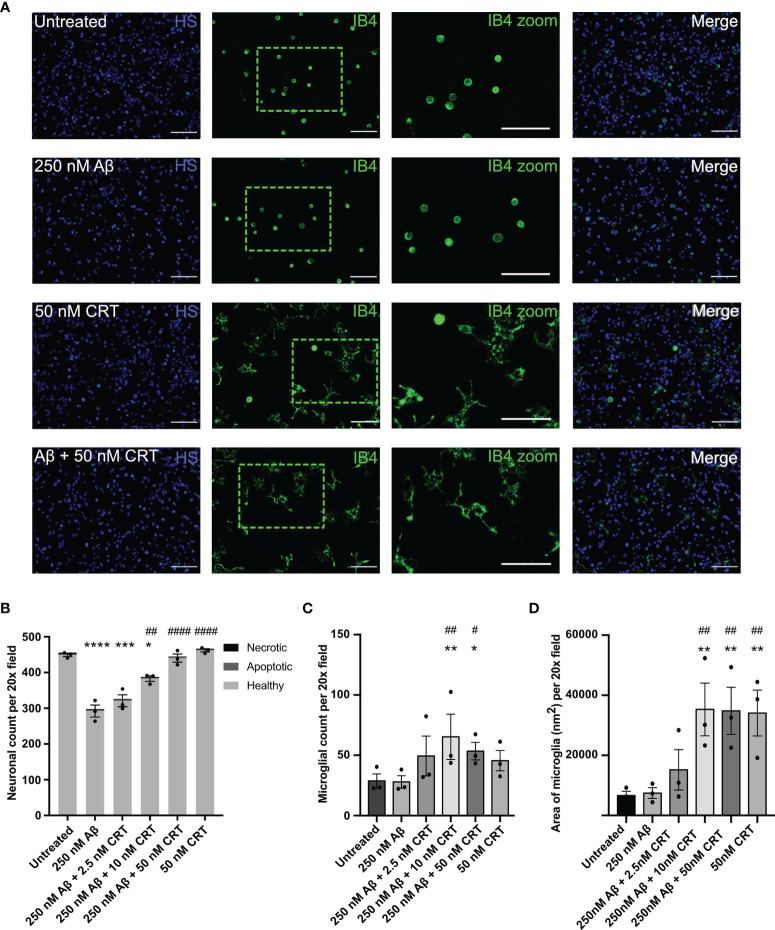
Calreticulin protects against amyloid beta induced neuronal loss. Mixed neuron-glial co-cultures were treated ± monomeric 250 nM Aβ and ± 2.5 nM, 10 nM or 50 nM calreticulin (CRT) for 72 h at 37 °C. **(A)** Cultures were stained with isolectin B4 (green, to identify microglia), propidium iodide (red, to identify necrotic cells, not shown), Hoechst 33342 (blue, to identify nuclei and apoptotic cells) and representative, imaged fields are shown. Scale bars = 100 µm. **(B)** Neuronal cell numbers and cell death were quantified by fluorescence microscopy. **(C)** Microglial cell numbers were quantified by fluorescence microscopy. **(D)** Microglial area was quantified as area of field stained with IB4. Data presented as mean neuronal count, microglial count, or area of microglia, with error bars representing the SEM of 3 independent experiments. Statistical comparisons were made by one-way ANOVA. Asterisk (*) indicate significance compared to untreated control (*p<0.05, **p<0.01, ***p<0.001, ****p<0.0001). Hash (#) indicate significance compared to Aβ only (^#^p<0.05, ^##^p<0.01, ^####^p<0.0001).

As calreticulin affects fibrillization and oligomerization of Aβ, we next tested whether calreticulin affected the neurotoxicity induced by Aβ. We have previously shown that nanomolar concentrations of Aβ induce neuronal loss mediated by microglia in mixed neuron–glial co-cultures (26). Here, we found that 250 nM Aβ induced loss of about 35% of the neurons over 72 hours (**Figures 7A, B**). 50 nM calreticulin alone (in the absence of Aβ) had no effect on neuronal or microglial numbers (**Figures 7A–C**), but did dramatically affect microglial morphology, as microglia became flatter with increased area (**Figures 7A, D**). Thus, calreticulin activates microglia in a way that is not detrimental to neurons. Indeed, addition of 10 nM calreticulin significantly inhibited the Aβ-induced neuronal loss, and 50 nM calreticulin almost completely prevented the Aβ-induced neuronal loss (**Figures 7A, B**). Thus, extracellular calreticulin is neuroprotective at concentrations similar those released by brain cells in a variety of circumstances.

## Discussion

We investigated here whether brains cells release calreticulin, and whether extracellular calreticulin had any effects on microglia and neurons relevant to neuroinflammation and neurodegeneration. We found that nanomolar levels of calreticulin were released from crushed primary neurons, apoptotic BV-2 microglia killed with staurosporine, and BV-2 under ER stress from tunicamycin. This extends our previous finding that LPS can induce calreticulin release from primary mouse microglia (13). As calreticulin can be released from neurons and microglia in a variety of conditions, we tested whether extracellular calreticulin could act as an alarmin. We found that nanomolar calreticulin can chemoattract microglia and activate microglia to release proinflammatory cytokines. This indicates that calreticulin can act an alarmin for microglia.

Alarmins are endogenous molecules released by necrotic cells and inflamed immune cells, to recruit and activate immune cells, in order to restore homeostasis of damaged tissues (14). Calreticulin is recognized as a damage-associated molecular pattern (DAMP), expressed on the surface of dead or dying cells, where it may induce phagocytosis and antigen presentation by dendritic cells [so called: immunogenic cell death (45, 46)]. These pro-phagocytic and immunogenic functions of calreticulin are dependent on being bound to the surface of dead or dying cells. However, calreticulin can also be released from activated neutrophils (47) and macrophages (12), and extracellular calreticulin can induce activation of monocytes, B cells and dendritic cells (15, 48). Thus, it is possible that calreticulin may act as an alarmin to immune cells other than microglia. Exogenous calreticulin has been shown to stimulate wound healing by recruiting macrophages, stimulating the proliferation of keratinocytes, fibroblasts and endothelial cells, and increasing extracellular matrix production (42). Thus, if calreticulin is endogenously released by damaged tissues in the body or brain, it may help repair that tissue.

We did not investigate the mechanism by which calreticulin activates microglia, other than to show that chelation of LPS by polymyxin B, or inhibition of LRP1 by LRPAP1, did not prevent calreticulin-induced activation of microglia, which suggests that the activation is not mediated by LPS or LRP1. However, it has previously been shown that nanomolar calreticulin can induce the release of soluble LRP1 (sLRP1) from microglia, and that sLRP1 alone can induce cytokine release by an unknown mechanism (49). So, it is possible that calreticulin binds to LRP1 to induce the release of sLRP1, which then induces cytokine release, although one might expect that LPRAP1 would block such a mechanism (49). Alternatively, as a proteolytic fragment of calreticulin has previously been shown to activate myeloid cells *via* TLR4/CD14 (50), it is possible that full-length calreticulin binds to TLR4 or CD14 to activate microglia.

We found that calreticulin affected Aβ aggregation. In conditions of Aβ oligomerization, calreticulin increased the formation of larger Aβ oligomers. In conditions of Aβ fibrillization, calreticulin folded Aβ into aggregates with less β-structure and Aβ fibrillization. This is consistent with calreticulin acting as an extracellular chaperone for Aβ. Within the ER, calreticulin is an intracellular chaperone, binding proteins with a hydrophobic surface, enabling refolding (51). Calreticulin can directly bind Aβ *via* the polypeptide binding site of calreticulin and the hydrophobic C-terminus of Aβ (21), and a significant fraction of Aβ in human cerebrospinal fluid is found bound to calreticulin (20), suggesting that extracellular calreticulin may significantly impact Aβ aggregation in human brain. It has previously been shown that five different chaperones: αB-crystallin, heat shock protein 70, clusterin, haptoglobin and α_2_-macroglobulin, each block Aβ neurotoxicity by binding to Aβ oligomers, promoting their assembly into larger oligomeric species, with consequent shielding of the reactive surfaces and reduced fibrilization (52, 53). This fits our data for calreticulin, therefore suggesting that calreticulin inhibits Aβ neurotoxicity by acting as an Aβ chaperone.

As calreticulin induced microglial activation and interfered with Aβ aggregation, we tested whether it affected Aβ-induced neurotoxicity. Nanomolar levels of calreticulin prevented Aβ-induced neurotoxicity. We did not investigate the mechanism of this protection, but possible mechanisms include calreticulin interfering with Aβ aggregation or microglial activation. Another possibility is that calreticulin increases microglial uptake and degradation of Aβ.

Calreticulin alone induced a strong morphological transition of microglia in primary neuron-glial co-cultures, from an unattached, spheroidal morphology to an attached and partially ramified morphology. This may appear contrary to the finding that microglial activation *in vivo* is accompanied by a transition from a highly ramified morphology to a more amoeboid morphology (30). However, in culture, untreated microglia are mainly spheroidal and relatively unattached (on uncoated culture plates), but become attached and flatten onto the culture plate in response to LPS (54, 55), and become ramified in response to ATP (55) or TNF-α and INFγ (54).

Calreticulin alone had no significant effect on microglial numbers in primary neuron-glial co-cultures, and microglial numbers increased in the presence of both calreticulin and Aβ, but calreticulin alone caused a small decrease in proliferation of BV-2 cells. BV-2 cells are an immortalized microglial cell line (37), so their proliferative response may not be representative of primary cells. Microglial activation generally increases proliferation (56). However, calreticulin has been found to inhibit the proliferation of a variety of cell types (40, 41, 57).

It used to be thought that microglia could be activated into only two main states, M1 and M2, but there are now known to be multiple forms of microglial activation (58, 59), some neurotoxic and some neuroprotective (60, 61). Calreticulin appears to recruit and activate microglia without neurotoxicity, consistent with calreticulin being an alarmin.

In summary, neurons and microglia can release calreticulin in multiple conditions, resulting in nanomolar levels of extracellular calreticulin. Nanomolar calreticulin can induce microglial migration and activation, and can interfere with Aβ aggregation and neurotoxicity. As calreticulin has been found in human cerebrospinal fluid (20), it may be protecting the brain by acting as an alarmin, extracellular chaperone and neuroprotectant. If so, increasing extracellular calreticulin levels might be beneficial in brain pathologies.

## Data Availability Statement

The original contributions presented in the study are included in the article/**Supplementary Material**. Further inquiries can be directed to the corresponding author.

## Ethics Statement

The animal study was reviewed and approved by University of Cambridge Animal Welfare and Ethical Review Body.

## Author Contributions

KR, EK, CB, and TC performed and analyzed experiments. GB conceived and managed the research. GB and KR wrote most of the manuscript. All authors reviewed and approved the manuscript.

## Funding

This work received funding from the Biotechnology and Biological Sciences Research Council UK (BBSRC BB/M011194/1).

## Conflict of Interest

The authors declare that the research was conducted in the absence of any commercial or financial relationships that could be construed as a potential conflict of interest.

## Publisher’s Note

All claims expressed in this article are solely those of the authors and do not necessarily represent those of their affiliated organizations, or those of the publisher, the editors and the reviewers. Any product that may be evaluated in this article, or claim that may be made by its manufacturer, is not guaranteed or endorsed by the publisher.
