# Supplementary material for: Brain Cells Release Calreticulin That Attracts and Activates Microglia, and Inhibits Amyloid Beta Aggregation and Neurotoxicity
Source: Front Immunol. 2022 Apr 20;13:859686. doi: 10.3389/fimmu.2022.859686 (PMC9065406; doi:10.3389/fimmu.2022.859686)
Supplement: Supplementary file 1 [file DataSheet_1.pdf]

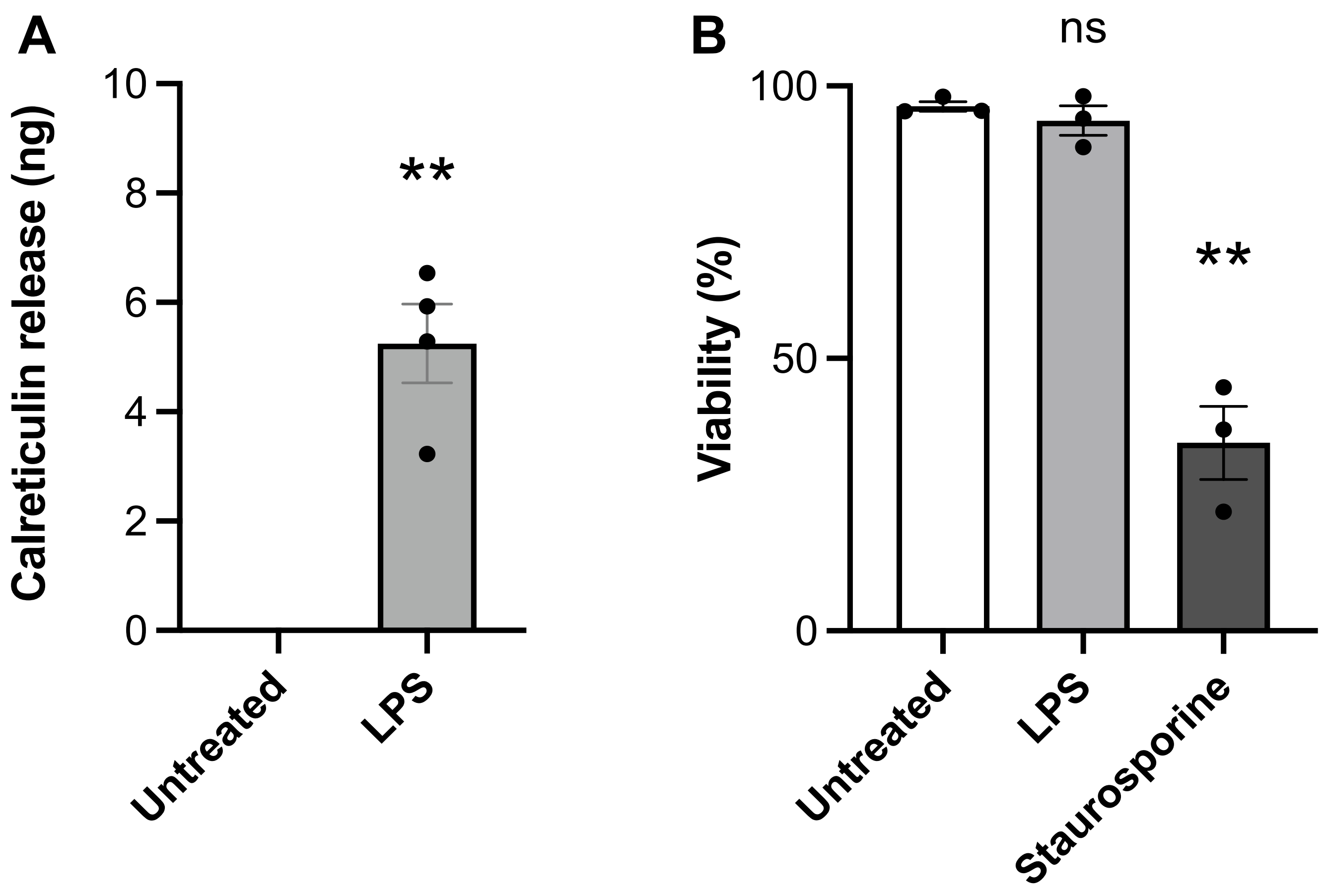

Supplementary Figure 1. **Calreticulin is released from inflamed BV-2 microglia.** (**A**) BV-2 microglia were treated with LPS (100 ng/mL) for 24 hours. Culture supernatants were then assessed for calreticulin release by ELISA. (**B**) Viability is the % of non-necrotic cells and was assessed by differential dye uptake (Hoechst/propidium iodide) and visualised by fluorescence microscopy, with cells treated for 6 hours with 1000 nM staurosporine acting as a positive indicator of cell death. The dataset represents the mean ± SEM of at least 3 independent experiments. Statistical comparisons were made by (**A**) paired t-test and (**B**) one-way ANOVA. ns: $p \geq 0.05$, **: $p < 0.01$.

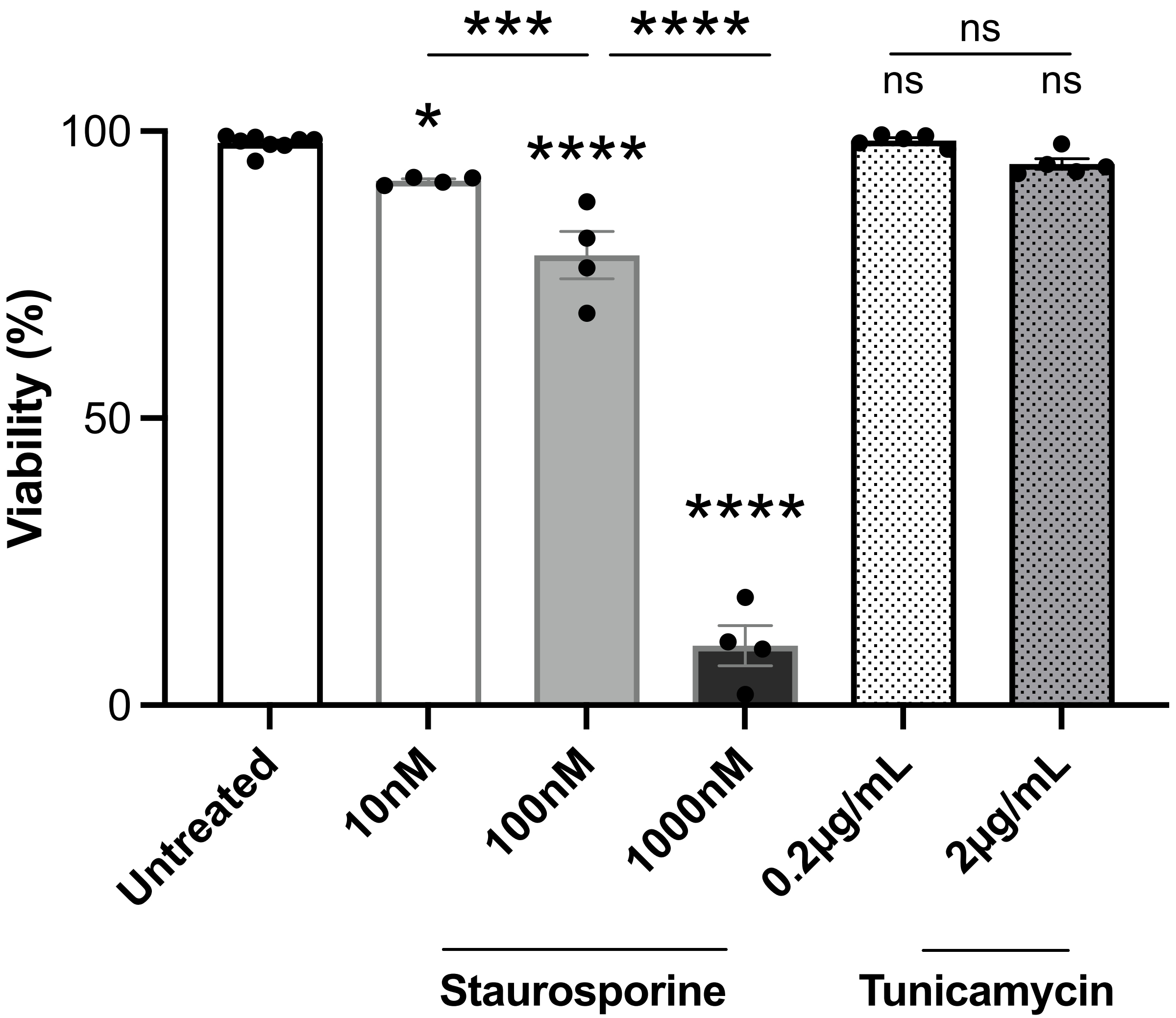

Supplementary Figure 2. **Viability of staurosporine and tunicamycin treated BV-2 microglia.** Viability of BV-2 microglia treated for 20 hours with staurosporine (0, 10, 100 and 1000 nM) and tunicamycin (0.2 and 2 µg/mL). Viability is the % of non-necrotic cells and was assessed by differential dye uptake (Hoechst/propidium iodide) and visualised by fluorescence microscopy. Each dataset represents the mean ± SEM of at least 3 independent experiments. Statistical comparisons were made to the untreated control, or as illustrated by a comparison line, by one-way ANOVA. ns: $p \geq 0.05$, *: $p < 0.05$, ***: $p < 0.001$, ****: $p < 0.0001$.

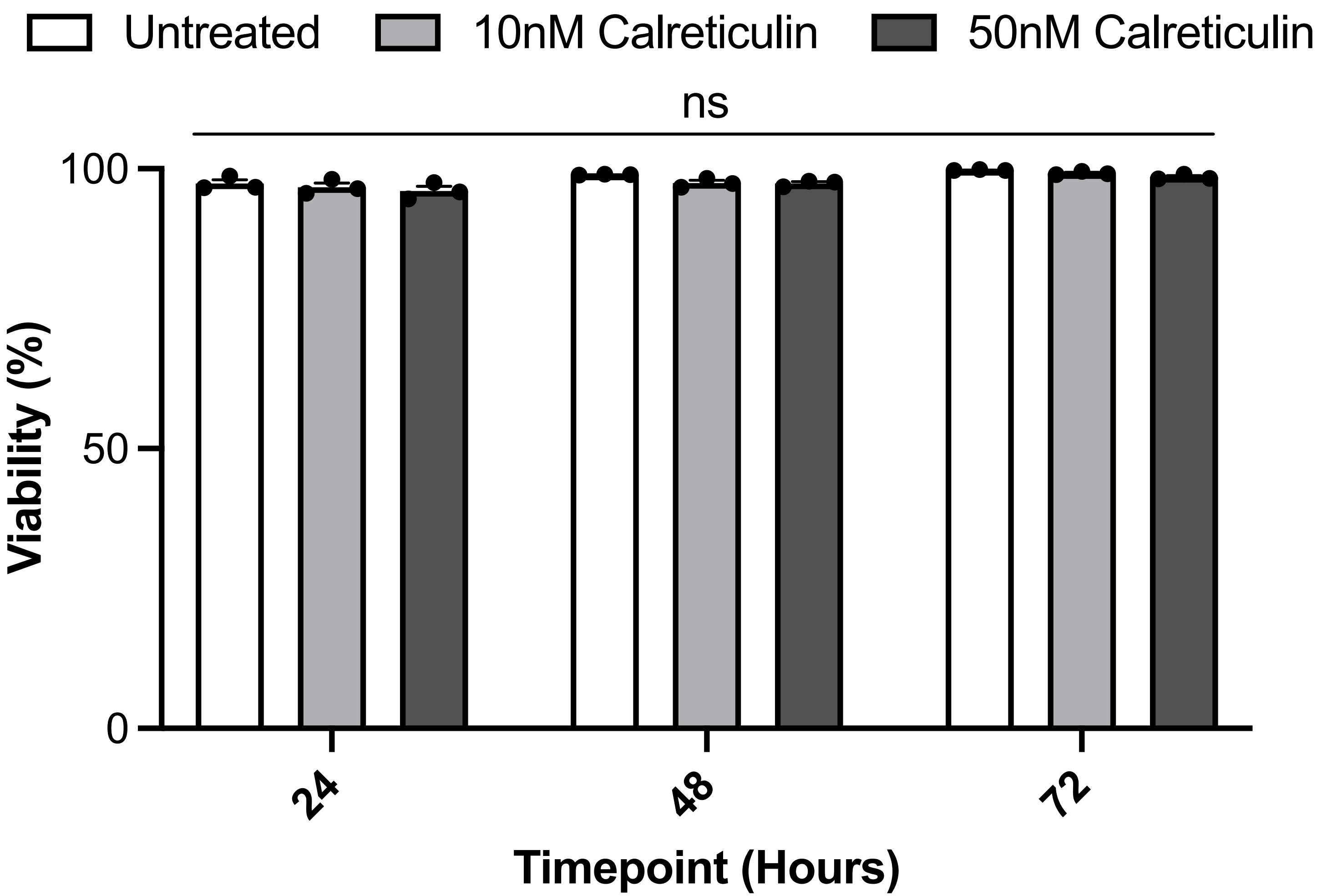

Supplementary Figure 3. **Calreticulin does not affect the long-term viability of BV-2 microglia.** Viability of BV-2 microglia treated for 24, 48 and 72-hour periods with 0, 10 or 50 nM calreticulin. Viability is the percentage of cells that were not necrotic, assessed by differential dye uptake (Hoechst 33342/propidium iodide) and visualised by fluorescence microscopy. Data presented as mean cell viability values, with error bars representing the SEM of 3 independent experiments. Statistical comparisons were made by two-way ANOVA. ns: $p \geq 0.05$.
